# Bilateral Inguinal Hernias Masking Cryptorchidism: A Case Report

**DOI:** 10.7759/cureus.64103

**Published:** 2024-07-08

**Authors:** Iman Moradi, Paige Finkelstein, Akil Paturi, Behrooz Shojai Rahnama, James O'Connor

**Affiliations:** 1 Medicine, St. George's University School of Medicine, St. George's, GRD; 2 General Surgery, Columbia University Business School, New York City, USA; 3 Orthopaedics, St. George's University School of Medicine, St. George's, GRD; 4 General Surgery, Icahn School of Medicine at Mount Sinai, Queens Hospital Center, Queens, USA

**Keywords:** multi-disciplinary surgical approach, testicular descent, testicular preservation, intraperitoneal testicle, inguinal herniorrhaphy, cryptorchidism

## Abstract

This case report discusses the uncommon occurrence of bilateral inguinal hernias masking cryptorchidism in a 47-year-old male, emphasizing the complexities and nuances of diagnosing and managing undescended testes in adults. Cryptorchidism is most often identified and treated during infancy; however, its rare adult manifestation presents significant diagnostic challenges and is fraught with serious implications for fertility and malignancy if left untreated. The subject presented with long-standing bilateral groin discomfort and was initially evaluated using clinical examination and CT imaging, which diagnosed bilateral inguinal hernias but did not initially detect cryptorchidism. During the staged surgical repairs, the testes were unexpectedly discovered within the hernia sacs, significantly altering the surgical approach and postoperative management. This necessitated an intraoperative consultation and collaboration between general surgery and urology, highlighting the critical role of multidisciplinary teamwork in managing complex surgical cases. The report underscores the importance of meticulous preoperative assessment and raises awareness about the potential for unusual findings in adult inguinal hernia repairs. This case report stresses the need for careful postoperative follow-up and regular urological surveillance to monitor for potential complications, including the development of testicular cancer. This case contributes valuable insights into the management strategies and long-term considerations for adult cryptorchidism, reinforcing the need for heightened clinical suspicion in similar presentations to ensure optimal patient outcomes.

## Introduction

Inguinal hernias are among the most common conditions managed by general surgeons, characterized by the protrusion of abdominal contents through the inguinal canal. They are predominantly found in males, with a lifetime risk of 27% in men compared to 3% in women [1]. Each year, more than 20 million inguinal hernia repairs are completed worldwide, including over 800,000 in the United States alone [2]. About 2-5% of indirect inguinal hernias are sliding, meaning part of the wall of the sac is formed by a visceral organ [3]. Inguinal hernias can sometimes contain unusual contents, such as omentum, bowel, or, urological organs.

Cryptorchidism is the failure of one or both testes to descend into the scrotal sac. It affects about 1-3% of male infants at one year of age and can lead to several complications if untreated, including infertility and an increased risk of testicular cancer [4,5]. While the majority of undescended testes are diagnosed and managed during childhood, adult presentations are rare and pose unique diagnostic and therapeutic challenges [6].

This case report presents the unique case of a patient who underwent bilateral hernia repair where the right testicle was found to be part of the hernia sac and the left testicle was free floating in the hernia sac. With intraoperative assistance from the urology service, the patient underwent two successful hernia repairs without harm to the testes.

## Case presentation

A 47-year-old married male immigrant, with no known medical or surgical history and limited access to healthcare in his country of origin, presented to the emergency department with a four-year history of bilateral groin discomfort. Physical examination was limited due to the patient's obesity, but notable scrotal distention was observed. Laboratory investigations, including CBC, a comprehensive metabolic panel (CMP), and a coagulation panel, were within normal limits except for mild normocytic anemia. No additional laboratory tests were performed. A CT scan revealed massive bilateral inguinal hernias extending into the scrotum, with the right side being larger than the left. The left inguinal hernia contained a nondilated bowel, while the right hernia included vessels with evidence suggesting vascular congestion. Additionally, bilateral scrotal hydroceles were present. He was diagnosed with bilateral indirect inguinal hernias. The patient was advised to follow up in the clinic to schedule an outpatient inguinal hernia repair. He returned to the clinic about two months later, and it was decided to repair the hernias in two separate operations. As the left hernia contained a small bowel, it would be repaired first. 

**Figure 1:**
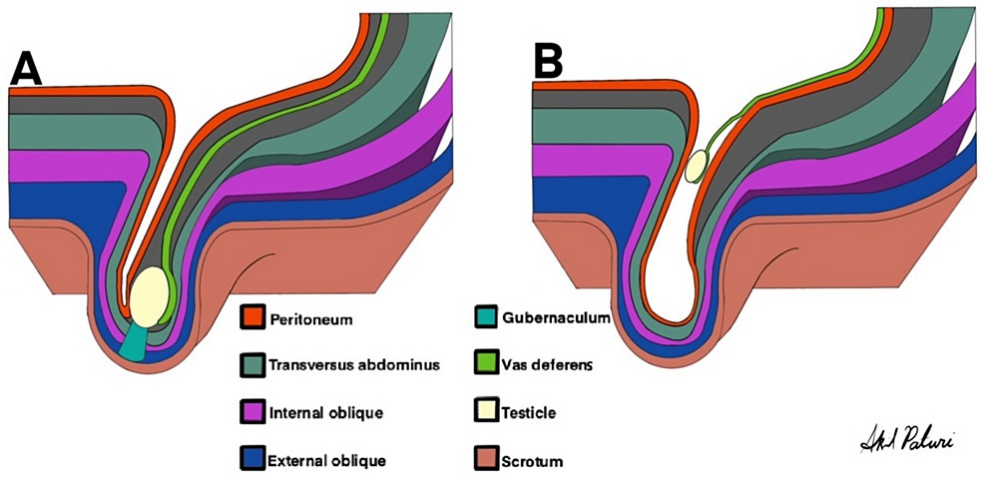
(A) Normally descended testis. (B) The patient’s testicle was atrophic and floating in the peritoneal hernia sac. Spermatic cords joined the peritoneum distal to the internal inguinal canal opening.

The plan was to perform a Lichtenstein repair of the left hernia. After mobilizing the cord structures off of the pubic tubercle, a direct hernia defect was not identified. Upon examining the cord structures, an indirect hernia sac was identified without any significant cord structures going distal to the hernia sac into the scrotum. The hernia sac was opened, and the testicle was found within the hernia sac with minimal adherence to the sac. The cord structures were adherent to the inside of the hernia sac just distal to the internal ring (Figure 1). Urology was consulted intraoperatively. A joint decision between urology and general surgery was made to return the testicle to the abdominal cavity. A mesh was placed over the inguinal canal without any cord structures within it, and the rest of the surgery proceeded as normal. The postoperative course was unremarkable. The surgical team decided to proceed with a Lichtenstein repair of the right side. In light of the atypical anatomy encountered during the initial surgery, the CT scan was reinterpreted. Given the concern for another ectopic testicle, urology would be available on standby.

**Figure 2:**
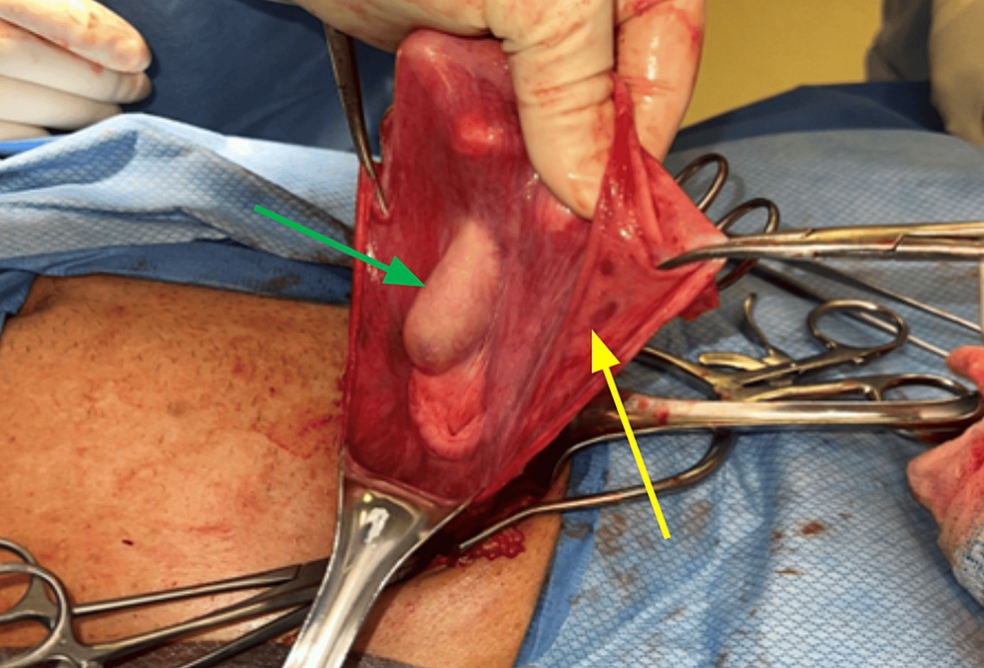
Intraoperative discovery of cryptorchidism. The yellow arrow points to the hernia sac. The green arrow points to the right testis, which is adherent to the peritoneal hernia sac.

Two months later, the patient presented to ambulatory surgery for right-sided inguinal hernia repair. As was previously seen during the left-sided hernia repair, the testicle or cord structures were not going through the inguinal canal into the scrotum. In contrast, the testicle was found to be adherent to the hernia sac (Figure 2). The hernia sac was ligated distal to the testicle, and the testicle was repositioned back into the preperitoneal space.

Two weeks postoperatively, the patient was seen in the clinic by both general surgery and urology and had no new complaints. Notably, the patient did not report any sexual dysfunction after the surgery.

## Discussion

**Figure 3:**
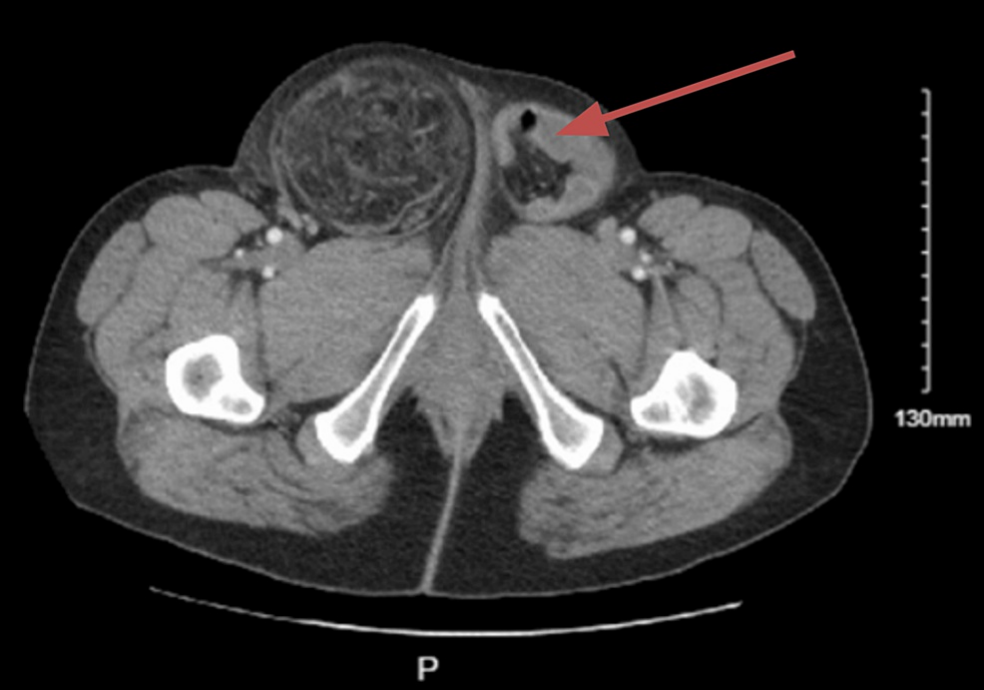
Preoperative CT scan. Bilateral inguinal hernias noted, left with bowel. Bilateral hydroceles are also present. The red arrow points to the testis visualized only after postoperative reinterpretation.

In evaluating inguinal hernias, a thorough testicular examination and CT imaging can be used to assess the possibility of cryptorchidism. Such information guides a coordinated multidisciplinary plan of care. A CT scan with intraoperative radiology reinterpretation enhances diagnostic accuracy, especially in complex or atypical cases such as this one [7,8]. However, the presence of large amounts of omental fat, small bowel, and hydroceles in the hernia hindered our ability to identify cryptorchidism on physical exams and imaging studies. As such, the initial CT scan interpretation did not identify the undescended testes, and only with an intraoperative reinterpretation were the testes visualized (Figure 3). During the operation, the testes and the cord structures were discovered to be inside the hernia sac on the left side, attached to the hernia sac on the right side, and undescended bilaterally. 

Cryptorchidism is a relatively common congenital abnormality. Prevalence of cryptorchidism at birth is reported to be 1.6-9%, but spontaneous descent leads to a decrease in rates to 1-3% by one year of age. Spontaneous descent leads to a persistent decrease in the prevalence of cryptorchidism up to puberty [9]. Testicular descent is believed to occur in two phases. The transabdominal phase is the movement of the testes across the lateral walls of the abdomen towards the inguinal canal. The transinguinal phase is the descent of the testes from the external ring to the scrotum. While recent advances in molecular biology have uncovered mechanisms responsible for these phases, the etiology of cryptorchidism remains elusive. However, in this case, the anatomical location of the testis suggests an arrest after the transabdominal phase [10].

A testis that remains undescended by puberty has a two to six times greater risk of developing testicular cancer. Although this risk is significantly lower than previously believed, it is still substantial enough for orchiectomy to be the treatment of choice for postpubertal cryptorchidism [3,11,12]. However, the management of postpubescent cryptorchidism is influenced by factors including risk of developing seminoma, the risk of anesthesia complications, and the patient’s wishes [13,14]. The patient did not receive an orchiectomy because he did not consent to an orchiectomy in the first surgery and did not wish for an orchiectomy in the second surgery. Intraoperative consultation with urology led to the decision that the patient would need to follow up with them for regular screening ultrasound for testicular cancer. Hence, he may require orchiectomies should complications be detected in regular surveillance.

A review of the literature has described case reports involving patients with sliding hernias containing epididymal cysts, atrophic testes, and unilateral cryptorchidism. However, Ansari et al. were the first to report a case of bilateral cryptorchidism associated with bilateral inguinal hernias [15].

## Conclusions

This case report illustrates the challenges encountered in complex inguinal hernia repairs, particularly when unexpected findings such as cryptorchidism arise. Thorough preoperative evaluation and interdisciplinary collaboration between general surgery and urology were essential in navigating this case. The identification of bilateral cryptorchidism underscores the importance of preoperative examination, especially in cases involving large hernias containing other intra-abdominal contents. The significance of postoperative follow-up and regular urological surveillance in patients with cryptorchidism cannot be overstated, given the increased risk of testicular cancer associated with undescended testes. This report highlights the necessity for effective communication between surgical specialties to ensure optimal outcomes in patients presenting with inguinal hernias and the associated anatomical anomalies. Further studies and clinical experiences may provide additional insights into the management and long-term implications of cryptorchidism in the adult population undergoing inguinal hernia repair.
